# Relationship between Plasma D-Dimer Concentration and Three-Dimensional Ultrasound Placental Volume in Women at Risk for Placental Vascular Diseases: A Monocentric Prospective Study

**DOI:** 10.1371/journal.pone.0156593

**Published:** 2016-06-13

**Authors:** Cécile Fanget, Céline Chauleur, Amandine Stadler, Emilie Presles, Marie-Noëlle Varlet, Jean-Christophe Gris, Tiphaine Raia-Barjat

**Affiliations:** 1 Department of Gynecology and Obstetrics, University Hospital, Saint Etienne, France; 2 Research Unit EA3065, Saint Etienne University Jean Monnet F 42023, Saint Etienne, France; 3 Laboratory of Haematology, University Hospital, Nîmes, France; 4 Research Unit EA2992, Montpellier University, Montpellier, France; St Francis Hospital, UNITED STATES

## Abstract

**Introduction:**

The aim of this study was to correlate placental volumes deduced from three-dimensional ultrasound and virtual organ computer-aided analysis (VOCAL) software with systemic concentrations of D-dimer and soluble endothelial protein C receptor (sEPCR).

**Methods:**

This was a monocentric experimental prospective study conducted from October 2008 to July 2009. Forty consecutive patients at risk of placental vascular pathology (PVP) recurrence or occurrence were included. Placental volumes were systematically measured three times (11–14, 16–18 and 20–22 weeks of gestation (WG)) by two independent sonographers. D-dimers and sEPCR plasma concentrations were measured using ELISA kits (Enzyme Linked ImmunoSorbent Assay).

**Results:**

Eleven patients had a PVP. The plasma D-dimer level was positively correlated with placental volume (*r* = 0.45, *p* < 0.001). A smaller placental volume and placental quotient was evidenced in women who developed a PVP at the three gestational ages, and the difference was more pronounced during the third exam (20 WG). No obvious correlation could be demonstrated between the development of a PVP and the levels of D-dimer and sEPCR. There was no significant difference in the values of placental volumes measured by the two sonographers.

**Conclusion:**

The placenta growth could be a major determinant of the elevation of D-dimer during pregnancy. Consideration of placental volume could allow for modulation of the D-dimer concentrations for restoring their clinical interest.

## Introduction

A placenta in pre-eclampsia (PE) or in intrauterine growth restriction (IUGR) is often the site of multiple infarcts that generate placental hypoperfusion [1–3]. The introduction of three-dimensional (3D) ultrasound techniques enables the placental volume up to 22 weeks of gestation (WG) to be determined quickly and easily. Some authors tried to find out whether or not the placentas of PE women or small-for-gestational age (SGA) foetuses have different growth characteristics compared to those of uncomplicated pregnancies. However, even if many studies found smaller placental volumes in PE women and SGA foetuses, this method alone is not able to predict a PVP [4–10]. It seemed interesting to us to evaluate the link between placental volume and two bioassays that increased during pregnancy or expressed by the placenta.

D-dimer levels increase progressively during a normal pregnancy, which alters their clinical utility in the diagnosis of venous thromboembolism. This increase is proportional with gestational age [11–13]. Some authors described higher levels of D-dimer in patients with IUGR, gestational hypertension, preeclampsia and *abruptio placentae* than in normal pregnancies [14–16]. The placenta growth could be a major determinant of the elevation of the D-dimer level during pregnancy but relation between placental volume and D-dimer levels has not been studied.

sEPCR (soluble endothelial protein C receptor) is an endothelial cell membrane glycoprotein that binds Protein C and activated Protein C (APC) [17–20]. sEPCR plays a critical role in foeto-maternal blood coagulation control and in preventing thrombosis at the maternal-embryonic interface [20]. The trophoblast constitutively expresses tissue factor and sEPCR so that sEPCR could be in relation with placental volume. The link between placental volume, plasma D-dimer and sEPCR levels and PVP is currently insufficiently documented.

The objective of this study was to correlate placental volume with systemic concentrations of D-dimers. The secondary objectives were to correlate placental volume with systemic concentrations of sEPCR; evaluate placental volume, plasma D-dimer and sEPCR levels for the prediction of placental vascular pathology in high-risk women; assess the feasibility of placental volume measurements deduced from 3D ultrasound scans at 12, 16 and 22 weeks of gestation; and determine the corresponding interobserver reproducibility.

## Materials and Methods

### Patients

This is a monocentric prospective longitudinal study carried out in our hospital. From October 2008 to July 2009, all women at 12 WG with a previous PVP or at risk of having a PVP because of a chronic vascular disease (hypertension, previous thromboembolic events or thrombophilic disorders) were included. The study was approved by the University Hospital of Saint Etienne institutional review board and ethics committee, and all subjects provided written informed consent. This clinical investigation was performed according to the Helsinki declaration of 1975, as revised in 1996. Exclusion criteria were multiple pregnancies and aneuploidies.

PVPs were defined by (1) fetal death ≥ 20 WG unrelated to fetal malformations, abnormal karyotype or infectious disease; (2) PE defined according to the International Society for the Study of Hypertension in Pregnancy criteria (ISSHP) as the occurrence of hypertension after 20 WG with a systolic ≥ 140 mmHg and/or diastolic ≥ 90 mmHg found on two measures, associated with proteinuria ≥ 0.3 g / 24 h or ≥ 1 + on dipstick 2 times without associated urinary tract infection; (3) eclampsia defined as the presence of convulsions in a preeclamptic patient; (4) *abruptio placentae* defined by the presence of externalised haemorrhage and/or found on pathologic analysis of placenta conducted in a context of preeclampsia, wood uterus or fetal distress; (5) Hemolysis, Elevated Liver enzyme and Low Platelet count (HELLP) syndrome defined by the combination of: hemolysis (LDH > 600 IU, bilirubin > 1.2 mg/dl, or presence of schizocytes in peripheral blood), elevated liver enzymes and platelets < 100,000; and (6) SGA defined by a birthweight ≤ to the 10^th^ customised centile.

Demographic data, medical history, pathologies during pregnancy, treatments and undesirable events were collected.

### Ultrasound

Placental volume and blood samples were performed at 11–14, 16–18 and 20–22 WG. The placental site was determined in 2D real-time mode using a transabdominal 3.5 MHz volume transducer (GE Voluson 730). The scanning plane was chosen at the place where the largest placental surface area was visualised. The placental volume was then scanned in this plane and saved; this procedure was repeated until a satisfactory image of the entire placenta was obtained [7]. The virtual organ computer aided analysis (VOCAL) technique was used to obtain a sequence of 6 sections of the placenta, each after a 30° rotation from the previous one. In each of the 6 planes, the contour of the placenta was drawn manually, which took approximately 60 seconds. The equipment automatically displayed the reconstructed image and its volume in cm^3^. During the second time, the placental volume was measured by a second sonographer (S2). The results of the first sonographer (S1) were unknown in order to compare the measurements and calculate intra- and interobserver agreements.

Measurements could not be performed on exactly the same day of gestation. Therefore we have calculated a quotient of placental volume divided by the number of gestational days (placental quotient [PQ] = placental volume/number of gestational days). Thus, we obtained 3 quotients: PQ1 (12 WG), PQ2 (16 WG) and PQ3 (20 WG) (8).

### Biology

All blood samples were collected in anticoagulated tubes, 9 volumes of blood for 1 volume of anticoagulant solution (0.109 M trisodium citrate).

Following a double centrifugation at 2500 *g* for 20 minutes, aliquots were snap frozen and immediately stored at -80°C. These aliquots were to be analysed in a single round at the end of the work. Laboratory workers were blinded to the results of placental volume measurements and to the development of a PVP. D-dimer and sEPCR concentrations were quantified by commercially available ELISA kits (Asserachrom, Stago, Asnières, France). Testing was performed in duplicate according to the manufacturer’s instructions in the Laboratory of Haematology, University Hospital, Nîmes, France.

### Statistical analysis

The different analyses were performed using the StatView^®^ software. Qualitative variables were reported as absolute and relative frequencies (expressed in %) and compared using Fisher’s exact test. Quantitative variables were described by mean and standard deviation and median and interquartile range and were compared by Student's *t*-test. In case of a non-normally distributed variable (assessed by a Shapiro-Wilk test), a Mann-Whitney U-test was performed. Group comparability between patients with a PVP and patients presenting no PVP was verified on the demographics and baseline characteristics.

We also used Spearman's rank-order correlation to elucidate a linkage between placental volume and D-dimer and between placental volume and sEPCR with their corresponding regression graphs.

A Wilcoxon rank-sum test was used to compare quantitative data between the different groups. The results were summarised in a box plot.

The non-parametric regression proposed by Passing and Bablock was used to estimate the relationship between placental volumes measured by the two observers. This linear regression procedure has no special assumptions regarding the distribution of the samples and the measurement errors. The result does not depend on the assignment of the methods (or instruments) to X and Y. The slope B and intercept A are calculated with a 95% confidence interval. These confidence intervals are used to determine whether or not there is only a chance difference between B and 1 and between A and 0. For all analyses, a statistical significance was established at *p* < 0.05.

## Results

Forty consecutive patients with eligible criteria were included from October 2008 to July 2009.

The patients’ characteristics and inclusion criteria are summarised in Table 1. Eleven PVP cases occurred. 8 neonates were SGA babies, 4 patients developed PE and one patient developed severe PE associated with SGA. No patient had thromboembolic issues. Demographic characteristics were not statistically different between patients who experienced a PVP (PVP+ group) and those who did not (PVP- group), except for gestational age at delivery (earlier for PVP+ group) and birth weight (lower in PVP+ group). Inclusion criteria were not statistically different between the two groups, except for history of fetal growth restriction, which was more frequent in the PVP+ group.

**Table 1:** Patients’ characteristics.

	Total n = 40		PVP—n = 29		PVP+ n = 11		P value
Demographic data: mean ± standard deviation median (Q1;Q3)	mean ± SD	median (Q1–Q3)	mean ± SD	median (Q1–Q3)	mean ± SD	median (Q1–Q3)	
Age (years)	31.5 ± 5.1	32.0 (27.5–35.5)	31.7 ± 5.6	32.0 (27.0–36.0)	30.9 ± 3.7	31.0 (28.3–33.8)	0.7
Gestity	2.8 ± 2.0	2.0 (1.0–4.0)	2.7 ± 2.0	2.0 (1.0–4.0)	3.1 ± 2.0	2.5 (2.0–3.75)	0.6
Parity	1.5 ± 1.1	1.0 (1.0–2.0)	1.4 ± 1.1	1.0 (1.0–2.0)	1.8 ± 1.1	2.0 (1.0–2.0)	0.4
BMI (Kg/m^2^)	24.8 ± 5.3	23.4 (21.1–27.9)	25.5 ± 5.6	23.2 (21.5–30.3)	22.6 ± 3.9	23.6 (19.3–24.6)	0.2
Term of delivery (WG)	38.4 ± 2.2	38.6 (37.7–39.6)	38.9 ± 1.9	39.2 (38.1–40.1)	36.9 ± 2.4	37.4 (36.6–38.3)	**0.006**
Birth weight (grams)	3025.7 ± 547.9	3072.5 (2815.0–3373.8)	3205 ± 393.4	3200 (2987,5–3497,5)	2487.8 ± 613.5	2645 (2380–2755)	**0.0004**
**Treatment:**	n	%	n	%	n	%	
Low-dose aspirin, 100 mg per day	17	42.5	11	37.9	6	54.5	0.5
Prophylactic LMWH 4,000 units per day	11	27.5	9	31.0	2	18.2	0.7
Two treatments	7	17.5	6	20.7	1	9.1	0.7
No treatment	5	12.5	3	10.3	2	18.2	0.6
**Inclusion criteria**	n	%	n	%	n	%	
Chronic hypertension	1	2.5	0	0	1	9.1	0.3
Systemic Lupus Erythematosus	2	5.0	2	6.9	0	0	1
Anti-phospholipid Syndrome	3	7.5	2	6.9	1	9.1	1
Venous thromboembolic history	11	27.5	9	31.0	2	18.2	0.7
Thrombophilia	5	12.5	4	13.8	1	9.1	1
History of PVP							
*Preeclampsia*	7	17.5	3	10.3	4	36.4	0.08
*Abruptio placentae*	2	5.0	2	6.9	0	0	1
*Vascular IUGR < 10th percentile*	4	10.0	0	0	4	36.4	**0.004**
*Fœtal death ≥ 10WG*	8	20.0	5	17.2	3	27.3	0.7
*At least 2 early miscarriages ≤ 10 WG*	10	25.0	9	31.0	1	9.1	0.2

**Fig 1:**
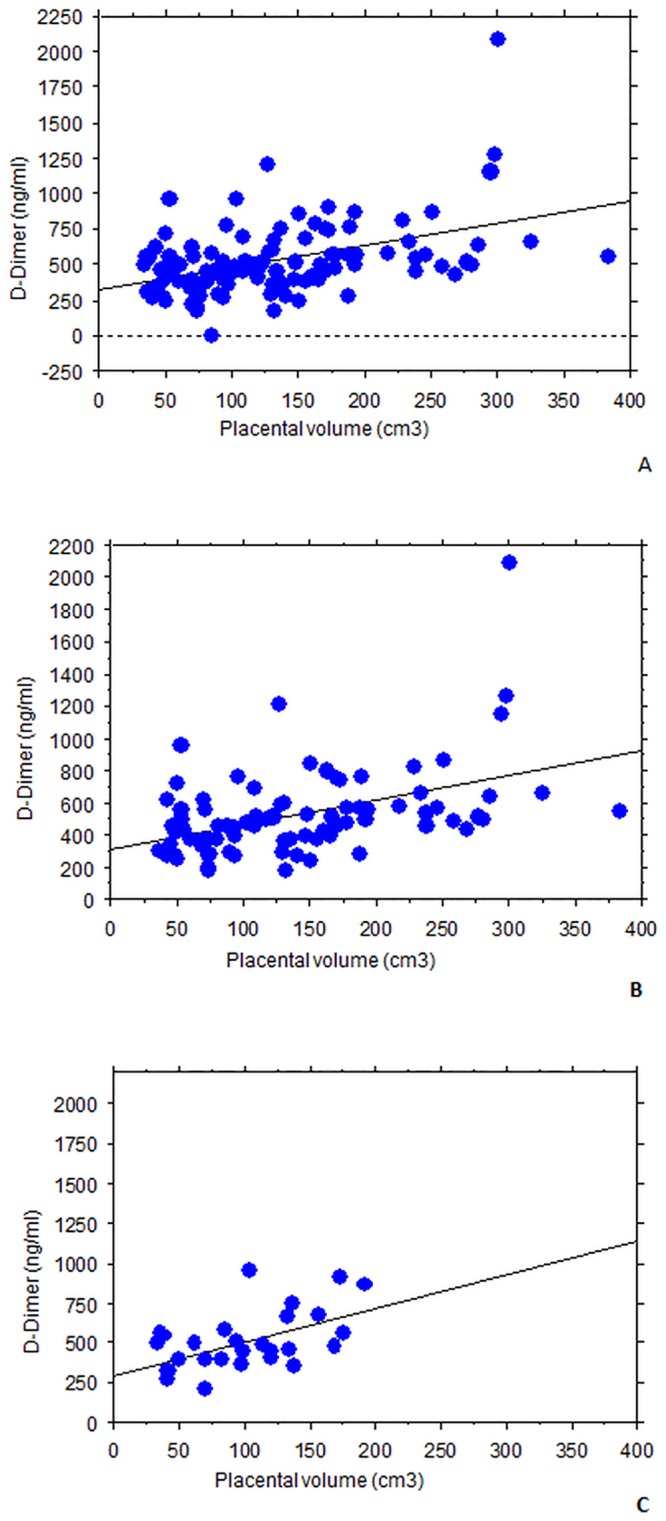
Plasma D-dimer level was positively correlated with placental volume. Regression graphs obtained from Spearman's rank-order correlation in order to elucidate linkage between placental volume and D-dimer. A. All patients and all measurements. B. Only patients with no PVP. C. Only patients with PVP.

**Fig 2:**
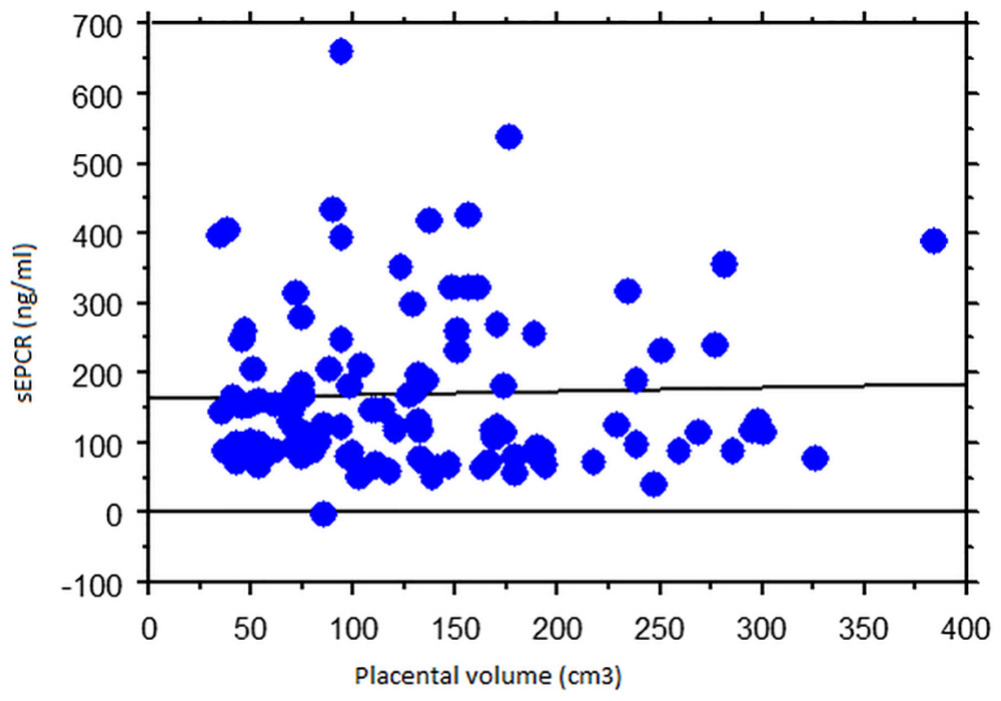
No relation between sEPCR and placental volume was found. Regression graphs obtained from Spearman's rank-order correlation in order to elucidate linkage between placental volume and sEPCR.

Plasma D-dimer level was positively correlated with placental volume (*r* = 0.45, *p* < 0.001; PVP- *r* = 0.45, *p* <0.001; PVP+ *r* = 0.49, *p* <0.001; Spearman rank test) (Fig 1). No relation between sEPCR and placental volume have emerged from our analysis (*r* = 0.03, *p* = 0.65; PVP- *r* = 0.07; PVP+ *r* = 0.02; Spearman rank test) (Fig 2).

We found a significant difference between placental volumes or PQ values according to the presence/absence of a PVP during the pregnancy under focus. Placental volumes were smaller in cases of PE or SGA, a difference that was significant as early as the first sonography (12 WG 61.5 cm^3^ for the PVP- group versus 51.0 cm^3^ for the PVP+ group, *p* = 0.04) but was more pronounced during the third exam (20 WG 231.8 cm^3^ for the PVP- group versus 155.8 cm^3^ for the PVP+ group, *p* = 0.004) (Table 2). We found no significant difference between plasma D-dimer levels values or plasma sEPCR level values according to the presence or absence of a PVP (Table 2).

**Table 2:** Ultrasound and blood samples results.

**Gestational window**	**12 weeks**		**16 weeks**		**20 weeks**	
**Placental Volume (cm**^**3**^)	mean ± SD	median (Q1–Q3)	mean ± SD	median (Q1–Q3)	mean ± SD	median (Q1–Q3)
Total	58.6 ± 18.0	53.0 (43.8–71.9)	127.9 ± 32.2	127.0 (99.6–148.9)	214.4 ± 65.2	191.0 (167.8–263.3)
PVP –	61.5 ± 17.7	54.2 (48.3–73.4)	133.9 ± 32.4	131.6 (109.1–151.6)	231.8 ± 63.4	237.0 (174.8–278.9)
PVP +	51.0 ± 17.3	41.0 (39.1–64.8)	112.8 ± 27.6	102.5 (95.2–125.6)	155.8 ± 25.6	162.0 (136.3–172.4)
P value		**0.04**		0.06		**0.004**
**Placental Quotient**	mean ± SD	median (Q1–Q3)	mean ± SD	median (Q1–Q3)	mean ± SD	median (Q1–Q3)
Total	0.68 ± 0.20	0.62 (0.51–0.83)	1.12 ± 0.29	1.09 (0.87–1.30)	1.51 ± 0.46	1.35 (1.17–1.87)
PVP –	0.71 ± 0.19	0.66 (0.59–0.85)	1.18 ± 0.29	1.18 (0.95–1.35)	1.36 ± 0.44	1.67 (1.23–1.96)
PVP +	0.59 ± 0.20	0.48 (0.44–0.74)	0.97 ± 0.23	0.90 (0.81–1.09)	1.08 ± 0.18	1.13 (0.95–1.19)
P value		**0.03**		**0.04**		**0.002**
**D-dimer (ng/ml)**	mean ± SD	median (Q1–Q3)	mean ± SD	median (Q1–Q3)	mean ± SD	median (Q1–Q3)
Total	411.7 ± 155.6	379.0 (298.5–505.5)	521.9 ± 203.1	482.0 (403.5–581.3)	648.4 ± 315.9	574.0 (484.0–712.5)
PVP –	413.3 ± 168.2	379.0 (294.0–461.0)	509.6 ± 209.7	482.0 (395.0–567.8)	665.4 ± 352.1	576.0 (495.0–744.0)
PVP +	407.2 ± 119.3	403.5 (329.3–507.5)	556.4 ± 189.3	481.5 (425.0–647.0)	599.1 ± 179.0	570.0 (481.5–665.0)
P value		0.92		0.54		0.82
**sEPCR (ng/ml)**	mean ± SD	median (Q1–Q3)	mean ± SD	median (Q1–Q3)	mean ± SD	median (Q1–Q3)
Total	164.0 ± 96.3	146.8 (94.4–180.5)	177.5 ± 132.7	127.3 (81.7–211.8)	168.8 ± 112.0	122.3 (90.3–224.1)
PVP –	157.1 ± 88.1	135.3 (93.3–188.7)	165.0 ± 108.8	128.1 (74.6–214.8)	166.1 ± 97.3	123.2 (91.0–235.3)
PVP +	183.9 ± 120.2	153.2 (108.0–164.6)	212.8 ± 187.0	127.3 (107.9–205.1)	177.7 ± 157.5	116.2 (89.7–148.7)
P value		0.53		0.42		0.68
**Gestational window**	**12 to 16 Weeks**		**16 to 20 Weeks**		**12 to 20 Weeks**	
**Placental Volume growth**	mean ± SD	median (Q1–Q3)	mean ± SD	median (Q1–Q3)	mean ± SD	median (Q1–Q3)
Total	132.9 ±74.5	126.8 (83.4–171.4)	67.9 ±45.1	64.7 (39.8–100.9)	285.7 ±126.5	282.7 (177.4–379.3)
PVP –	129.7 ±72.0	117.9 (84.7–154.6)	73.8 ±46.7	69.0 (47.7–107.1)	292.3 ±126.1	282.7 (213.1–379.3)
PVP +	141.2 ±83.7	142.9 (88.3–190.4)	48.5 ±35.2	43.1 (33.2–68.9)	263.5 ±134.0	270.8 (144.7–348.2)
P value		0.67		0.15		0.58

**Fig 3:**
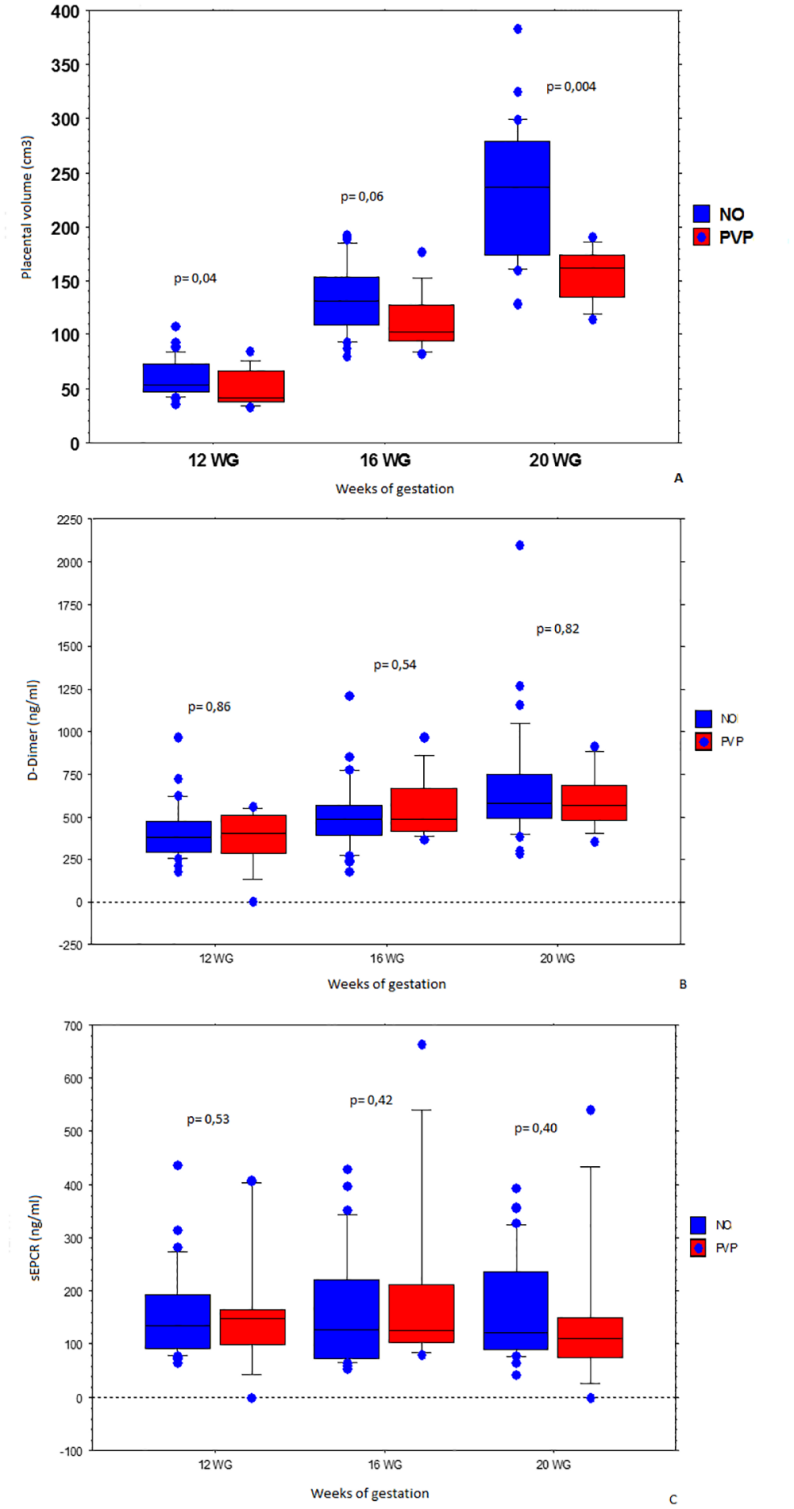
Placental volumes were smaller in cases of PVP. Summary with box plots of the distribution of values according to gestational age and the occurrence or not of a PVP. (A) Placental volume at 12, 16 and 20 WG and comparison of data based on the occurrence or not of a PVP. (B) D-dimer concentrations at 12, 16 and 20 WG and comparison of data based on the occurrence or not of a PVP. (C) sEPCR concentrations at 12, 16 and 20 WG and comparison of data based on the occurrence or not of a PVP.

The distributions of the placental volume, plasma D-dimer levels and sEPCR levels are presented as box plots in Fig 3. The mean placental volume was 58.6 cm^3^ for S1 versus 61.3 for S2 at 12 WG, 127.9 cm^3^ versus 132.5 at 16 WG and 214.4 cm^3^ versus 200.7 at 20 WG. The mean PQ value was 0.7 for S1 versus 0.71 for S2 at 12 WG (*p* = 0.68), 1.1 versus 1.2 at 16 WG (*p* = 0.57) and 1.5 versus 1.4 at 20 WG (*p* = 0.38).

With a Passing-Bablock linear regression analysis, the agreement between placental volumes or PQ measured by the two observers was excellent. The slope and intercept were not significantly different from 1 and 0, respectively.

## Discussion

We proved that the plasma D-dimer level was positively correlated with placental volume. To our knowledge, this is the only study that investigated this relationship. The value of D-dimer during pregnancy is higher than normal in 15% of patients in the 1st trimester, 71% in the second trimester and 96% in the third trimester [21]. Recent studies have tried to establish new reference intervals of D-dimer levels in pregnant women according to trimester. Reference intervals of D-dimer were determined by Ercan *et al*. as 110–400 ng/mL, 140–750 ng/mL and 160–1300 ng/mL in first, second and third trimester, respectively [22]. Therefore, other studies have tried to establish new thresholds of D-dimer references for each trimester to predict the risk of thrombosis in pregnant women. Nishii *et al*. studied the correlation between venous Doppler ultrasound of the lower limbs and D-dimer levels for the detection of venous thromboembolism disease (VTE) in a population of asymptomatic patients [11]. The authors showed that in the third trimester of pregnancy, the average value of the D-dimer positive ultrasound group was significantly higher than in the negative group. They concluded that D-dimer levels could not be used alone for screening VTE; however, it would be interesting to perform an ultrasound in pregnant women with D-dimer levels greater than 3200 ng/ml in the third trimester and would improve the asymptomatic VTE detection rate.

Many studies have shown that D-dimer levels are raised in women with PE compared to a normotensive pregnancy [23–25]. Ho *et al*. suggested that D-dimer levels could be used as complementary predictors for the development of PE [26]. In our study, we did not find any relationship between D-dimer and PVP development.

Because birthweight and placental weight at the time of delivery are closely correlated [6,27,28], some authors aimed to predict foetal growth restriction through placental volumes [4,10,27,29,30]. Although our study dealt with only forty patients, our results are in agreement with these studies. We found smaller volumes, especially at 20 WG but also at 12 WG in cases of PVP. Our findings are in contrast to those of Odeh *et al*. who reported that placental volume was not a predictor of PE developed after 34 WG and of SGA [31].

Wegrzyn *et al*. were the first to investigate placental volume using the VOCAL technique [32]. We used a 30° rotation and a sequence of 6 planes.

Hurtado *et al*. found that very high levels of IgM and IgG anti-sEPCR constituted a strong risk factor for the first episode of foetal death, with a 20-fold increase in relative risk [17]. Isermann *et al*. showed a new function for the thrombomodulin-protein C system in controlling the growth and survival of trophoblast cells in the placenta; this function is essential for the maintenance of pregnancy [33]. The hypercoagulable state developed during pregnancy may be increased in PVP probably due to the multiple infarcts in the placenta, and sEPCR seems to be a marker of endothelium activation/damage [34]. In our study, we did not find any relationship between sEPCR and PVP development.

One of the aims of the study was to determine interobserver reproducibility of placental volume measurements and its feasibility. This study shows that this technique is easy, quick (about 1 minute) and reproducible. The placental volumes are not significantly different between the two sonographers. Others studies assessed the reproducibility of placental volumes [3,5,35–38]. PQ in our study are quite similar with those of an unselected group of 1199 women: PQ1 0.66 vs 0.601, PQ2 1.14 vs 1.136 and PQ3 1.48 vs 1.643 [8]. The advantage of this method compared to second trimester uterine Doppler is that placental volume can be performed as early as the first trimester. Rizzo [10] and Hafner [4] found that a low placental volume in the first trimester is strongly associated with an abnormal uterine Doppler in the second trimester and that both have similar sensitivities for predicting PE; however, there is no relationship between placental volume and uterine Doppler in the first trimester [9]. However, placental volume measurements are sometimes difficult to perform. In our study, the volume differences between the two sonographers are higher at 20 WG. It can be explained by the irregular shape of the placenta; from week 19 onward, the size of the placenta often exceeds the capacity of the equipment [6,7,39]. Despite this, the agreement between placental volumes measured by the two observers was very good.

## Conclusion

The plasma D-dimer level was positively correlated with placental volume. The placenta growth could be a major determinant of the elevation of the D-dimer level during pregnancy. Consideration of placental volume could then allow modulating D-dimer concentrations for restoring their clinical interest. All study data are available (S1 Table).

Placental volume is decreased in high-risk patients who experienced a PVP compared to patients who did not have a PVP at an early stage. Placental volume could complement of a PVP screening strategy.

## Supporting Information

S1 Table All available data. (PDF)
