# Supplementary material for: Relationship between Plasma D-Dimer Concentration and Three-Dimensional Ultrasound Placental Volume in Women at Risk for Placental Vascular Diseases: A Monocentric Prospective Study
Source: PLoS One. 2016 Jun 13;11(6):e0156593. doi: 10.1371/journal.pone.0156593 (PMC4905670; doi:10.1371/journal.pone.0156593)
Supplement: S1 Table — (PDF) [file pone.0156593.s001.pdf]

N°	EVE / Oui-No	PVP	TTT	EVALUATION	SA	J	VP1	QP1	VP2	QP2	D-Di	sEPCR
20	NON	NON	OUI	12 WG	11	4	48,460	0,598	50,270	0,621	422,000	158,188
34	NON	NON	TTT-NON	12 WG	11	5	68,010	0,829	50,420	0,615	335,000	135,280
8	EVE-OUI	PVP-OUI	TTT-NON	12 WG	11	6	40,190	0,484	38,090	0,459	329,000	166,100
36	NON	NON	OUI	12 WG	11	2	36,110	0,457	34,000	0,430	311,000	91,600
22	EVE-OUI	PVP-OUI	OUI	12 WG	11	6	32,710	0,394	39,070	0,471	508,000	401,570
30	EVE-OUI	PVP-OUI	OUI	12 WG	11	6	49,000	0,590	47,000	0,566	405,000	102,950
31	NON	NON	OUI	12 WG	11	2	52,110	0,660	56,080	0,710	505,000	99,160
33	NON	NON	OUI	12 WG	11	6	43,000	0,518	57,000	0,687	303,000	78,409
27	NON	NON	OUI	12 WG	11	0	46,000	0,597			461,000	262,370
38	EVE-OUI	PVP-OUI	OUI	12 WG	12	5	69,000	0,775	68,200	0,766	402,000	123,200
10	EVE-OUI	PVP-OUI	OUI	12 WG	12	2	85,040	0,989	90,980	1,058	0,000	0,000
23	EVE-OUI	NON	OUI	12 WG	12	3	69,000	0,793	52,860	0,608	379,000	148,791
32	NON	NON	OUI	12 WG	12	1	50,240	0,591	94,390	1,110	722,000	207,990
17	NON	NON	TTT-NON	12 WG	12	1	44,000	0,518	40,170	0,473	346,000	248,000
16	NON	NON	OUI	12 WG	12	6	48,290	0,537	52,350	0,582	273,000	88,430
15	EVE-OUI	PVP-OUI	OUI	12 WG	12	2	41,000	0,477	43,340	0,504	330,000	73,824
2	NON	NON	OUI	12 WG	12	1	60,000	0,706			383,000	93,295
25	NON	NON	OUI	12 WG	12	2	74,000	0,860	95,690	1,113	279,000	188,680
13	NON	NON	OUI	12 WG	12	5	44,500	0,500	46,970	0,528	346,000	153,180
11	NON	NON	OUI	12 WG	12	1	78,980	0,929	72,820	0,857	374,000	93,295
26	NON	NON	OUI	12 WG	12	2	53,200	0,619	55,820	0,649	967,000	72,770
14	EVE-OUI	NON	OUI	12 WG	12	0	50,080	0,596	56,100	0,668	251,000	98,159
28	NON	NON	OUI	12 WG	12	3	89,200	1,025	81,280	0,934	294,000	437,820
5	NON	NON	OUI	12 WG	12	1	93,000	1,094			446,000	248,190
19	EVE-OUI	PVP-OUI	OUI	12 WG	12	3	60,600	0,697			506,000	159,640
6	NON	NON	OUI	12 WG	12	0	70,000	0,833			621,000	172,280
4	EVE-OUI	PVP-OUI	OUI	12 WG	12	3	69,750	0,802			218,000	97,106
1	NON	NON	OUI	12 WG	12	0	52,700	0,627			557,000	164,480
3	NON	NON	OUI	12 WG	12	2	73,450	0,854			379,000	83,563
29	NON	NON	TTT-NON	12 WG	12	4	54,210	0,616	61,030	0,694	441,000	95,539
18	NON	NON	OUI	12 WG	12	0	73,350	0,873	79,900	0,951	211,000	283,470
40	NON	PVP-OUI	OUI	12 WG	12	0	35,400	0,421	30,500	0,363	558,000	146,770
7	EVE-OUI	PVP-OUI	OUI	12 WG	12	3	38,140	0,438	37,880	0,435	545,000	407,210
21	NON	NON	OUI	12 WG	12	1	41,000	0,482	57,660	0,678	280,000	83,563
39	NON	NON	OUI	12 WG	12	0	73,010	0,869	78,000	0,929	183,000	116,860
35	NON	NON	OUI	12 WG	13	1	42,330	0,460			618,000	101,050
9	NON	NON	OUI	12 WG	13	4	107,900	1,136	94,940	0,999	458,000	65,703
12	NON	NON	OUI	12 WG	13	0	74,770	0,822			284,000	170,718
37	EVE-OUI	PVP-OUI	OUI	12 WG	13	0	40,000	0,440			271,000	160,210
24	NON	NON	OUI	12 WG	14	3	71,470	0,708	113,800	1,127	557,000	315,480

VP1-12	QP1-12	VP2-12	QP2-12	DDi-12	sEPCR-12	SA-16	J-16	VP1-16	QP1-16	VP2-16	QP2-16	VP1*16-VP1*
48,460	0,60	50,270	0,62	422,000	158,188	16	0	191,000	1,71	196,310	1,75	142,540
68,010	0,83	50,420	0,61	335,000	135,280	16	5	109,000	0,93	95,000	0,81	40,990
40,190	0,48	38,090	0,46	329,000	166,100	16	6	119,880	1,02	125,140	1,06	79,690
36,110	0,46	34,000	0,43	311,000	91,600	16	0	80,000	0,71	94,000	0,84	43,890
32,710	0,39	39,070	0,47	508,000	401,570	16	6	92,430	0,78	84,560	0,72	59,720
49,000	0,59	47,000	0,57	405,000	102,950	16	3	119,000	1,03	105,000	0,91	70,000
52,110	0,66	56,080	0,71	505,000	99,160	16	0	131,100	1,17	139,080	1,24	78,990
43,000	0,52	57,000	0,69	303,000	78,409	16	1	146,460	1,30	160,260	1,42	103,460
46,000	0,60			461,000	262,370	16	0	169,320	1,51	194,970	1,74	123,320
69,000	0,78	68,200	0,77	402,000	123,200	16	5	85,400	0,73			16,400
85,040	0,99	90,980	1,06	0,000	0,000	16	6	176,430	1,50	217,000	1,84	91,390
69,000	0,79	52,860	0,61	379,000	148,791	16	0	134,920	1,20	140,930	1,26	65,920
50,240	0,59	94,390	1,11	722,000	207,990	16	5	127,000	1,09	175,700	1,50	76,760
44,000	0,52	40,170	0,47	346,000	248,000	16	1	93,000	0,82	108,600	0,96	49,000
48,290	0,54	52,350	0,58	273,000	88,430	16	2	109,980	0,96	101,750	0,89	61,690
41,000	0,48	43,340	0,50	330,000	73,824	16	6	98,310	0,83	99,300	0,84	57,310
60,000	0,71			383,000	93,295	16	0	188,220	1,68	162,950	1,45	128,220
74,000	0,86	95,690	1,11	279,000	188,680	16	4	192,550	1,66	170,540	1,47	118,550
44,500	0,50	46,970	0,53	346,000	153,180	16	3	109,190	0,95	117,740	1,02	64,690
78,980	0,93	72,820	0,86	374,000	93,295	16	1	164,840	1,46	159,070	1,41	85,860
53,200	0,62	55,820	0,65	967,000	72,770	16						
50,080	0,60	56,100	0,67	251,000	98,159	16	0	93,130	0,83	83,360	0,74	43,050
89,200	1,03	81,280	0,93	294,000	437,820	16	0	156,000	1,39	145,170	1,30	66,800
93,000	1,09			446,000	248,190	16	6	122,680	1,04	117,570	1,00	29,680
60,600	0,70			506,000	159,640	16	2	102,450	0,90	88,860	0,78	41,850
70,000	0,83			621,000	172,280	16	3	150,000	1,30	155,000	1,35	80,000
69,750	0,80			218,000	97,106	16	3	82,580	0,72	85,230	0,74	12,830
52,700	0,63			557,000	164,480	16	0	150,060	1,34	156,780	1,40	97,360
73,450	0,85			379,000	83,563	16	1	178,000	1,58	156,220	1,38	104,550
54,210	0,62	61,030	0,69	441,000	95,539	16	2	100,950	0,89	126,960	1,11	46,740
73,350	0,87	79,900	0,95	211,000	283,470	16	5	87,680	0,75	116,130	0,99	14,330
35,400	0,42	30,500	0,36	558,000	146,770	16	2	131,300	1,15	149,000	1,31	95,900
38,140	0,44	37,880	0,44	545,000	407,210	16	6	135,530	1,15	116,960	0,99	97,390
41,000	0,48	57,660	0,68	280,000	83,563	16	1	140,100	1,24	165,270	1,46	99,100
73,010	0,87	78,000	0,93	183,000	116,860	16	0	132,000	1,18	125,700	1,12	58,990
42,330	0,46			618,000	101,050	16	2	96,000	0,84	92,800	0,81	53,670
107,900	1,14	94,940	1,00	458,000	65,703	16	0	116,220	1,04	98,900	0,88	8,320
74,770	0,82			284,000	170,718	16	1	130,850	1,16	155,400	1,38	56,080
40,000	0,44			271,000	160,210	16	2	97,910	0,86	105,000	0,92	57,910
71,470	0,71	113,800	1,13	557,000	315,480	16	6	147,730	1,25	147,210	1,25	76,260

QP1*16-QP1	VP2*16-VP2*	QP2*16-QP2	D-Di-16	DDi16-12	sEPCR-16	sEPCR16-12	SA-20	J-20	VP1-20	QP1-20	VP2-20	QP2-20
1,107	146,040	1,132	509,000	87,000	75,447	-82,741	20	0	217,210	1,55	190,470	1,36
0,102	44,580	0,197	695,000	360,000	149,320	14,040	20	5	172,040	1,19	172,100	1,19
0,532	87,050	0,602	450,000	121,000	122,460	-43,640	20	5	168,680	1,16	163,460	1,13
0,257	60,000	0,409	455,500	144,500	111,790	20,190	20	0	167,000	1,19	174,000	1,24
0,389	45,490	0,246	513,000	5,000	663,320	261,750	20	4	174,610	1,21	193,600	1,34
0,444	58,000	0,347	418,000	13,000	127,010	24,060	20	5	114,000	0,79	115,000	0,79
0,511	83,000	0,532	613,000	108,000	132,100	32,940	20	0	168,950	1,21	155,250	1,11
0,778	103,260	0,731	405,000	102,000	72,144	-6,265	20	1	258,580	1,83	254,200	1,80
0,914			459,000	-2,000	270,300	7,930	20	6	234,000	1,60	238,160	1,63
-0,045			584,000	182,000	127,650	4,450	20	5	171,600	1,18	174,400	1,20
0,506	126,020	0,781					20	6				
0,412	88,070	0,651	383,000	4,000	193,510	44,719	20	3	238,050	1,66	225,400	1,58
0,494	81,310	0,391	1210,000	488,000	169,200	-38,790	20	4	294,000	2,04	155,000	1,08
0,305	68,430	0,488	403,000	57,000	397,821	149,821	20	3	188,000	1,31	194,690	1,36
0,428	49,400	0,311	478,000	205,000	68,952	-19,478	20	3				
0,356	55,960	0,338	446,000	116,000	88,430	14,606	20	3	137,080	0,96	126,160	0,88
0,975			772,000	389,000	94,917	1,622	20	5	297,610	2,05	314,750	2,17
0,799	74,850	0,357	566,000	287,000	70,480	-118,200	20	2	177,490	1,25	151,630	1,07
0,449	70,770	0,496	512,000	166,000	151,560	-1,620	20	6				
0,530	86,250	0,551	399,000	25,000	75,447	-17,848	20	0	268,110	1,92	250,660	1,79
							20	4	300,000	2,08		
0,235	27,260	0,076	271,000	20,000	124,070	25,911	20	4	187,030	1,30	175,700	1,22
0,368	63,890	0,362	382,000	88,000	429,480	-8,340	20	0	383,000	2,74	390,780	2,79
-0,054			516,000	70,000	353,800	105,610	20	4	280,760	1,95	283,500	1,97
0,202			965,000	459,000	213,000	53,360	20	1				
0,471			852,000	231,000	261,030	88,750	20	3	250,000	1,75	223,000	1,56
-0,084			406,000	188,000	103,020	5,914	20	3	134,000	0,94	120,630	0,84
0,712			241,000	-316,000	235,340	70,860	20	0	227,540	1,63		
0,721			573,000	194,000	59,156	-24,407	20	6	325,110	2,23	282,900	1,94
0,270	65,930	0,420	486,000	45,000	54,328	-41,211	20	0	285,000	2,04	256,490	1,83
-0,124	36,230	0,041	462,000	251,000	208,000	-75,470	20	2	128,190	0,90	117,170	0,83
0,730	118,500	0,944	668,000	110,000	80,918	-65,852	20	2	191,000	1,35	202,000	1,42
0,710	79,080	0,556	751,000	206,000	420,640	13,430	20	3	155,360	1,09	137,200	0,96
0,757	107,610	0,784	280,000	0,000	67,328	-16,235	20	1	237,000	1,68	220,820	1,57
0,309	47,700	0,194	180,000	-3,000	120,030	3,170	20	1	129,000	0,91	144,000	1,02
0,382			773,000	155,000	84,057	-16,993	20	2	162,000	1,14	150,700	1,06
-0,098	3,960	-0,116	509,000	51,000	64,079	-1,624	20	1	246,370	1,75	261,750	1,86
0,336			361,000	77,000	201,560	30,842	20	1	277,010	1,96	244,730	1,74
0,419			363,000	92,000	181,440	21,230	20					
0,544	33,410	0,121	524,000	-33,000	323,470	7,990	20	3	160,150	1,12	164,010	1,15

VP1*20-VP1*	QP1*20-QP1'	VP2*20-VP2*	QP2*20-QP2'	D-Di-20	DDi20-16	sEPCR20-16	sEPCR-20	EVE / Oui-Nc	PVP.2	TTT.2	VP1*20-VP1*	QP1*20-QP1'
26,210	-0,154	-5,840	-0,392	582	73,000	1,624	77,071	NON	NON	OUI	168,750	0,953
63,040	0,255	77,100	0,375	744	49,000	33,410	182,730	NON	NON	TTT-NON	104,030	0,357
48,800	0,147	38,320	0,067	479	29,000	-15,957	106,503	EVE-OUI	PVP-OUI	TTT-NON	128,490	0,679
87,000	0,479	80,000	0,404	510	54,500	6,970	118,760	NON	NON	OUI	130,890	0,736
82,180	0,429	109,040	0,628	566	53,000	-121,410	541,910	EVE-OUI	PVP-OUI	OUI	141,900	0,818
-5,000	-0,249	10,000	-0,120	489	71,000	21,680	148,690	EVE-OUI	PVP-OUI	OUI	65,000	0,196
37,850	0,036	16,170	-0,133	757	144,000	-8,900	123,200	NON	NON	OUI	116,840	0,547
112,120	0,538	93,940	0,385	495	90,000	18,827	90,971	NON	NON	OUI	215,580	1,316
64,680	0,091	43,190	-0,110	662	203,000	51,340	321,640	NON	NON	OUI	188,000	1,005
86,200	0,454			913	329,000	-11,420	116,230	EVE-OUI	PVP-OUI	OUI	102,600	0,408
								EVE-OUI	PVP-OUI	OUI		
103,130	0,460	84,470	0,318	543	160,000	-3,320	190,190	EVE-OUI	NON	OUI	169,050	0,872
167,000	0,956	-20,700	-0,425	1159	-51,000	-47,900	121,300	NON	NON	OUI	243,760	1,451
95,000	0,492	86,090	0,400	576	173,000	-140,001	257,820	NON	NON	TTT-NON	144,000	0,797
				385	-93,000	25,021	93,973	NON	NON	OUI		
38,770	0,125	26,860	0,041	351	-95,000	-32,477	55,953	EVE-OUI	PVP-OUI	OUI	96,080	0,482
109,390	0,372	151,800	0,716	1268	496,000	38,863	133,780	NON	NON	OUI	237,610	1,347
-15,060	-0,410	-18,910	-0,402	479	-87,000	14,705	85,185	NON	NON	OUI	103,490	0,389
				609	97,000	14,460	166,020	NON	NON	OUI		
103,270	0,456	91,590	0,383	431	32,000	42,153	117,600	NON	NON	OUI	189,130	0,986
				2099			118,130	NON	NON	OUI	246,800	1,465
93,900	0,467	92,340	0,476	285	14,000	-36,362	87,708	EVE-OUI	NON	OUI	136,950	0,703
227,000	1,343	245,610	1,495	554	172,000	-35,750	393,730	NON	NON	OUI	293,800	1,710
158,080	0,910	165,930	0,972	503	-13,000	3,190	356,990	NON	NON	OUI	187,760	0,856
				617	-348,000	-213,000	0,000	OUI	PVP-OUI	OUI		
100,000	0,444	68,000	0,212	873	21,000	-25,690	235,340	NON	NON	OUI	180,000	0,915
51,420	0,219	35,400	0,102	454	48,000	-27,573	75,447	EVE-OUI	PVP-OUI	OUI	64,250	0,135
77,480	0,285			820	579,000	-108,030	127,310	NON	NON	OUI	174,840	0,998
147,110	0,652	126,680	0,555	659	86,000	19,538	78,694	NON	NON	OUI	251,660	1,373
184,050	1,150	129,530	0,718	641	155,000	35,724	90,052	NON	NON	TTT-NON	230,790	1,420
40,510	0,153	1,040	-0,167	599	137,000	93,090	301,090	NON	NON	OUI	54,840	0,030
59,700	0,193	53,000	0,116	867	199,000	8,795	89,713	NON	PVP-OUI	OUI	155,600	0,924
19,830	-0,062	20,240	-0,032	681	-70,000	-95,570	325,070	EVE-OUI	PVP-OUI	OUI	117,220	0,648
96,900	0,441	55,550	0,104	458	178,000	30,831	98,159	NON	NON	OUI	196,000	1,198
-3,000	-0,264	18,300	-0,101	301	121,000	54,320	174,350	NON	NON	OUI	55,990	0,046
66,000	0,299	57,900	0,247	798	25,000	-18,792	65,265	NON	NON	OUI	119,670	0,681
130,150	0,710	162,850	0,973	570	61,000	-22,762	41,317	NON	NON	OUI	138,470	0,612
146,160	0,807	89,330	0,360	516	155,000	40,210	241,770	NON	NON	OUI	202,240	1,143
				574	211,000	-41,690	139,750	EVE-OUI	PVP-OUI	OUI		
12,420	-0,132	16,800	-0,101	420	-104,000	3,200	326,670	NON	NON	OUI	88,680	0,412

VP2*20-VP2*	QP2*20-QP2*	DDi20-12	sEPCR20-12
140,200	0,740	160,000	-81,117
121,680	0,572	409,000	47,450
125,370	0,668	150,000	-59,597
140,000	0,812	199,000	27,160
154,530	0,874	58,000	140,340
68,000	0,227	84,000	45,740
99,170	0,399	252,000	24,040
197,200	1,116	192,000	12,562
		201,000	59,270
106,200	0,436	511,000	-6,970
172,540	0,969	164,000	41,399
60,610	-0,034	437,000	-86,690
154,520	0,889	230,000	9,820
		112,000	5,543
82,820	0,378	21,000	-17,871
		885,000	40,485
55,940	-0,045	200,000	-103,495
		263,000	12,840
177,840	0,934	57,000	24,305
		1132,000	45,360
119,600	0,552	34,000	-10,451
309,500	1,857	260,000	-44,090
		57,000	108,800
		111,000	-159,640
		252,000	63,060
		236,000	-21,659
		263,000	-37,170
		280,000	-4,869
195,460	1,139	200,000	-5,487
37,270	-0,126	388,000	17,620
171,500	1,059	309,000	-57,057
99,320	0,524	136,000	-82,140
163,160	0,888	178,000	14,596
66,000	0,093	118,000	57,490
		180,000	-35,785
166,810	0,857	112,000	-24,386
		232,000	71,052
		303,000	-20,460
50,210	0,020	-137,000	11,190
